# Management of Strategic and Operational Plans in Rescue Organizations to Maintain Preparedness against Disasters

**Published:** 2012-06-30

**Authors:** A H Faghih, M R Ebrahimi, M Abbasi

**Affiliations:** 1 President of Iranian Red Crescent Society, Tehran, Iran; 2 Director of Fars Red Crescent Society, Shiraz, Iran; 3 Advisor of President and Director General of the President Department of Iranian Red Crescent Society, Tehran, Iran

**Keywords:** SWOT-analysis, Management, Strategic plan, Rescue, Disaster, Iran

Iranian Red Crescent Society is a charitable and non-profit organization playing an active role in public-benefiting and humanistic affairs and services offered for rescue operations. The prospect of this society has been mentions as Red Crescent Society should be accountable for all kind of events in all parts of Iran with the highest possible efficiency and with minimal time required for services.[1] In the back twenty years, over three millions lives and at least 800 millions people have been affected by natural disasters all over the world.[2] The frequency of encountering disasters in developing and underdeveloped countries has been increased more than other countries due to rapid population growth, increasing urbanization, economic inequality, and climate change.[3] Iran has always faced a variety of natural disasters as well as road accidents. In recent years, many people have died in earthquakes. According to statistics, the primary cause of death in Iran was reported to be road accidents.[4] Disasters often occur unexpectedly, so they are always in the need of an immediate reaction. When responding to a disaster and determining the most appropriate action, there may be a critical delay and waste of time in taking an action. Therefore, ambiguity in cooperation control of cycles and lack of transparency when making decisions during a crisis may lead to confusion and a reduction in effectiveness of an appropriate response. A great number of countries and communities do not have a comprehensive plan to cope disasters. All countries should have comprehensive preparation plans to deal with different incidents.[5] Therefore, planning and adopting an efficient strategy against a possible accident is regarded as a necessity for organizations responsible for disasters such as Red Crescent Society. Employing proper strategies in health care organizations is regarded as one of important management strategies in many developed countries, especially SWOT (strengths, weaknesses, opportunities, threats)-analysis that seems to be more general than other analyses. However, researches on the use of this management tool are not very common.[6] One of the experiences of using SWOT analysis has been done by Deputy of Rescue and Assistant of Fars Red Crescent Society resulted into a long-term 3 years plan and a one year operational action. One of the benefits of SWOT analysis is that it can be used for planning in these organizations which can be implemented with the full participation of managers and experts and therefore to strengthen motivation to increase participation and implement strategies.[7] This participation and motivation reinforcement can be observed clearly in Fars Red Crescent Society that apply SWOT analysis from 10 months ago. SWOT analysis was defined based on the following criteria: (i) Strengths: Internal qualities of an organization helpful to achieving organizational goals, (ii) Weaknesses: Internal qualities of an organization harmful in achieving organizational goals, (iii) Opportunities: External conditions helpful to achieving organizational goals and (iv) Threats: External conditions harmful to achieving organizational goals.[8][9]

SWOT as a guide can analyze factors affecting the competition between the organizations and companies while providing a prospect for the future.[10] Therefore, to get prepared for coping accidents and disasters, the employment of a strategic and operational program based on SWOT analysis is considered as a rational decision in order to be aware of the present situation and anticipating future activities in rescue organizations and making these organizations up to date.
